# Neonicotinoid exposure causes behavioral impairment and delayed mortality of the federally threatened American burying beetle, *Nicrophorus americanus*

**DOI:** 10.1371/journal.pone.0314243

**Published:** 2025-01-21

**Authors:** Michael C. Cavallaro, Michelle L. Hladik, R. Shane McMurry, Samantha Hittson, Leon K. Boyles, W. Wyatt Hoback

**Affiliations:** 1 Department of Entomology and Plant Pathology, Oklahoma State University, Stillwater, OK, United States of America; 2 California Water Science Center, U.S. Geological Survey, Sacramento, CA, United States of America; 3 School of Life Sciences, University of Nevada Las Vegas, Las Vegas, NV, United States of America; University of Carthage, TUNISIA

## Abstract

Among the most immediate drivers of American burying beetle (*Nicrophorus americanus* Olivier) declines, nontarget toxicity to pesticides is poorly understood. Acute, episodic exposure to neonicotinoid insecticides at environmentally relevant concentrations is linked to negative impacts on beneficial terrestrial insect taxa. Beyond mortality, behavioral indicators of toxicity are often better suited to assess sublethal effects of residual concentrations in the environment. First, *Nicrophorus* spp. congeners were used to generate and identify a low-dose exposure rate (lethal dose 10%; LD10) from an acute, 24-hour exposure and the concentration-series was confirmed by LC–MS/MS. Next, we evaluated the effects of single and repeated low-dose (LD10 = 58.9 ng/beetle) imidacloprid exposure on *N*. *americanus* behavior (10 minutes post-dose) and mortality (10 days post-dose). Behavior parameters were analyzed using EthoVision-XT. Control *N*. *americanus* were significantly less mobile, demonstrating death-feigning, an anti-predator behavior. Single LD10 dosed *N*. *americanus* were hyperactive, traveling over 4 times farther (total distance; *p* = 0.03) and faster (mean velocity; *p* = 0.02) than controls. Single and repeated LD10 dosed *N*. *americanus* extended their wings without taking flight and flipped on their backs. All control *N*. *americanus* survived 10 days post-dose; single LD10 and repeated LD10 exhibited 30% and 50% mortality, respectively. A single LD10 exposure event was sufficient to significantly elicit greater movement and high predation risk behaviors, whereas repeated LD10 exposure did not worsen behavioral impairment but increased mortality over time. Collectively, generalized linear mixed effects models indicated that distance traveled, velocity, and extended wings were significant predictors of mortality. Recently reclassified, the federally threatened *N*. *americanus* may be at greater risk to insecticide exposure than previously thought and vulnerable to episodic, low-dose neonicotinoid exposure.

## Introduction

Broad-spectrum neonicotinoid insecticides represent the largest market share of any insecticide class at 27% worldwide with at least 140 crop uses [1]. Specifically, neonicotinoid-coated seed treatments are the most widely adopted crop protection strategy, especially for common field crops [2]. However, it is estimated that greater than 90% of the neonicotinoid active ingredient applied could move from the site of application via runoff and/or particulate matter [3]. Field monitoring of residual neonicotinoid concentrations in air, water, soil, and nontarget plant tissue collected peripheral to conventional agricultural operations confirms the high mobility of neonicotinoids in the environment [4–7]. Low-dose, episodic exposure is reported among nontarget insects and induces both sublethal effects and mortality, if exposure is prolonged [8].

Pesticide risk assessment routinely uses acute mortality data to evaluate toxicity to terrestrial insects; however, test concentrations often exceed environmentally relevant amounts. Chronic, sublethal levels can result in a wide range of detrimental behavioral effects at the population-level, which may increase mortality from predation in natural settings [9]. Behaviors associated with feeding, predator avoidance, mobility, sensory perception, and navigation are among the most affected by neurotoxic compounds, as they are linked to insect neurophysiology and biochemistry. Sublethal, behavioral effects of neonicotinoid exposure to insect pollinators are well-documented [10–12]. Comparatively few studies report the impacts of neonicotinoids, both lethal and sublethal endpoints, on nontarget beetle taxa. These studies have primarily focused on ladybird and ground beetles [13,14], with recent studies adding fireflies and dung beetles [15,16].

Burying beetles in the subfamily Nicrophorinae (Coleoptera: Silphidae) perform important ecosystem services by burying carrion, increasing available nutrients in soil, and expediting carrion decomposition, while acting as a food source for secondary consumers [17–20]. Seasonal abundance of the genus *Nicrophorus* will vary among diurnal, crepuscular, and nocturnal species that partition ephemeral carrion resources. Regardless of diel activity patterns, all species spend extended periods of time interacting with and manipulating surface and subsurface soils [21]. Because of their unique life history strategy [21,22], sensitivity to habitat change [23], distinct anti-predator and parental behaviors [24], and response to standardized trapping methods [25], burying beetles are an extensively studied taxon. Despite numerous laboratory- and field-based studies, there are substantial data gaps associated with the potential threats posed by current-use insecticides, like neonicotinoids, to burying beetles. To our knowledge, no neonicotinoid toxicity data exist for burying beetles, potentially resulting in inadequate protection of *N*. *americanus* in North America [26] at-risk *Nicrophorus* spp. in Europe, i.e., *N*. *antennatus*, *N*. *germanicus*, and *N*. *sepultor* [27].

In the United States, numerous studies on *Nicrophorus* have supported life history research and conservation efforts for extant populations of the federally threatened American burying beetle, *N*. *americanus* Olivier [28]. Once common across eastern North America, *N*. *americanus* distribution has reduced by more than 90%, remaining in only six states with the largest populations concentrated in Oklahoma and Nebraska [29]. The U.S. Fish and Wildlife Service (USFWS) first listed *N*. *americanus* as an endangered species in 1989 [30]; however, the USFWS recently reclassified *N*. *americanus* as threatened in 2020 [31], citing a diminished threat of extinction in its current range. Current and historical causes of *N*. *americanus* decline include habitat loss to agricultural and urban development, changes in the availability of carrion resources (birds and small mammals), light pollution, and pesticide use [28]. USFWS [32] provides predicted scenarios of *N*. *americanus* population resiliency that consider variable projected rates of land use conversion and climate change. The total area of insecticide use is predicted to increase as a result of shifts in land use and climate change [33]. However, concurrent risks of nontarget pesticide exposure to *N*. *americanus* were not included in the Species Status Assessment [32]. In a recent biological evaluation conducted by the U.S. Environmental Protection Agency (USEPA), *N*. *americanus* range was categorized as having “high” overlap with neonicotinoid occurrence [34]. Although pesticide exposure was considered an underlying factor of *N*. *americanus* declines [35], no study has directly assessed toxicity of any insecticide active ingredients to *N*. *americanus*, or other *Nicrophorus* species.

Here, we assessed the effects of single and repeated low-dose (lethal dose 10% mortality; LD10) imidacloprid exposure on *N*. *americanus* behavior and movement parameters (10 minutes post-dose) and mortality (10 days post-dose). Acknowledging the conservation status of *N*. *americanus*, *Nicrophorus* spp. congeners were used first to generate imidacloprid lethal dose (LD_X_) values because of their shared life history traits and physiology. Imidacloprid was selected because of its continued detection in the environment, the similar mode of action compared to other neonicotinoid active ingredients, and the existing extensive body of research on nontarget insects [13,14], including beetles of conservation concern [36]. We hypothesized that the LD10 imidacloprid dose would have measurable effects on *N*. *americanus* behavior and movement. Accordingly, repeated LD10 exposure would induce a greater behavioral response and higher mortality. We also hypothesized a comparable response in 24-hour contact toxicity between *N*. *americanus* and *Nicrophorus* spp. congeners.

## Materials and methods

### Burying beetle collection and husbandry

Adult burying beetles were collected using buried (Nebraska) or aboveground (Oklahoma) pitfall traps baited with carrion and followed modified protocol by Bedick et al. [25] and Leasure et al. [37]. *Nicrophorus marginatus* F. and *Nicrophorus carolinus* L. were collected near O’Neill, Nebraska. *Nicrophorus americanus* were collected at Camp Gruber located near Braggs, Oklahoma and were used as a part of a maintained *N*. *americanus* breeding program at Oklahoma State University (Permit no. TE-045150-4.1). Burying beetles were transported to Oklahoma State University in 5-gallon (18.9 L) buckets with moistened soil. Wild-caught *N*. *marginatus* and *N*. *carolinus* were sorted into separate plastic containers and fed *ad libitum* on 50/50 ground beef/pork until testing. Wild-caught *N*. *americanus* were introduced to brood buckets and allowed to breed following procedures outlined in McMurry et al. [38].

### Dose-response contact toxicity

Technical grade imidacloprid (>98% purity) was purchased from Sigma Aldrich (St. Louis, Missouri, USA). Imidacloprid was dissolved in analytical grade acetone in a nominal concentration series and was applied in 1 μL of acetone dispensed on the abdomen of each beetle using a 10 μL microsyringe (Hamilton; Reno, Nevada). Four nominal concentrations were used: 0 (acetone-only), 60, 120, 240 ng/μL. Topical applications were administered to 30 individuals per concentration for a total of 120 beetles. To compensate for the sampling constraints of using wild-caught beetles, 60/40 mix *N*. *marginatus* and *N*. *carolinus* were used to generate acute *Nicrophorus* spp. LD_X_ values. Beetles were weighed (g), dosed, and placed in individual plastic cups with no food. Burying beetles are prone to desiccation and were supplied with a moist cotton ball [25]. Mortality was assessed 24-hours after application by placing each beetle on its back; a beetle was considered dead if it did not flip over after 2 minutes.

### Imidacloprid-induced behavior and mortality

Described *Nicrophorus* spp. imidacloprid contact toxicity tests generated an acute (24-hour) *Nicrophorus* spp. LD10 dose, which was used for subsequent testing with the threatened *N*. *americanus*. Three LD10 imidacloprid treatment groups were used to assess the effect on *N*. *americanus*: control (acetone-only), single LD10 dose, and repeated LD10 dose (two separate LD10 doses 48 hours apart). Similar to the methods above, the LD10 dose was applied in 1 μL of acetone. Topical application was administered to 10 individuals per treatment group for a total of 30 *N*. *americanus*. These 30 adult *N*. *americanus* were selected from a synchronous lab colony (F1 offspring) with individuals subjected to toxicity testing 19 days after eclosion. Weight (g), sex, and size measured by pronotal width (mm) were recorded for each beetle.

For behavioral assays, treated *N*. *americanus* were singly introduced in the center of a standardized circular arena with a diameter of 18.4 cm and recorded using a smartphone camera (4K HDR video) for 10 minutes post-dose. Movement parameters were analyzed using EthoVision-XT (Noldus, Wageningen, The Netherlands). All videos were manually assessed for tracking errors. Movement parameters included total distance (mm), mean velocity (mm/sec), mobility % (percent of non-overlapping area occupied by the detected beetle that changes between samples), and mobility status (immobile: <20%, mobile: 20% - 60%, highly mobile: >60%). Beetle behavior was manually assessed for each video and focused on two frequently exhibited behaviors: total time (sec) beetle wings were extended without taking flight and total time (sec) each beetle was on its back. Movement and behavioral parameters were extracted from EthoVision-XT or manually documented per second over the 10-minute observation period (i.e., 600 time-series observations). After the 10-minutes observation period, *N*. *americanus* were individually separated in plastic containers (7.6 cm L×7.6 cm W×7.8 cm H) with a moist paper towel and fed two mealworms, *Tenebrio molitor* L., and two greater waxworm larvae, *Galleria mellonella* L. every 48–72 hours for 10 days. Mortality was assessed daily as above.

### Chemical analysis: Imidacloprid-spiked acetone

Imidacloprid acetone solutions (*n* = 3 per dose group) were measured by directly injecting, or diluting and then injecting, 10 μL onto an Agilent Technologies (Santa Clara, California) 1260 infinity bio-inert high-performance liquid chromatograph coupled to a 6430 triple quadrupole mass spectrometer (LC–MS/MS). Dilutions were made using acetonitrile. Instrument details and parameters are described elsewhere [39]. The instrument limit of detection (LOD) was 0.0025 ng/μL.

### Data analyses

Statistical analyses were conducted in R version 4.2.3 [40]. Topical *Nicrophorus* spp. LD_X_ values and their 95% confidence intervals were determined using generalized linear models (GLMs package *stats*) with a binomial distribution (logit link). Measured imidacloprid concentrations were log-transformed. Threshold LD_X_ concentrations were calculated using the dose.p function (package *MASS*; [41]), and dose is described as ng of imidacloprid each beetle received for each treatment. Two different species were used to generate *Nicrophorus* spp. LD50, LD20, and LD10 values; a similar model structure was used to determine the potential interaction effect between beetle weight or species and dose.

Measured *N*. *americanus* movement and behavioral parameters were analyzed using linear models with three treatment levels (i.e., acetone control, single LD10 dose, and repeated LD10 dose), followed by a Type II ANOVA (package *car*; [42]) and pairwise comparisons (ɑ  =  0.05) using posthoc Tukey tests (package *emmeans*; [43]). Results of posthoc comparisons are given on the log odds ratio, not the response scale [43]. Following the same approach, we also evaluated the number of food items (mealworm and waxworm larvae) consumed per feeding event, which was corrected for relative number of feedings per beetle. Data were subjected to normality (package *stats*) and variance (package *car*; [42]) checks by Kolmogorov–Smirnov and Levene’s test, respectively, as required.

A series of generalized linear mixed models (GLMMs package *lme4*; [44]) were used to determine relative sources of variation in behavior and movement parameters that resulted in 10-day *N*. *americanus* mortality. The binary response variable (i.e., *N*. *americanus* mortality) was assessed with a binomial distribution (logit link). The global model set was evaluated by the dredge function (package *MuMIn*; [45]). Fixed effects included: total distance (mm) each beetle traveled by treatment, mean velocity (mm/sec) calculated from each beetle by treatment, total time (sec) beetle wings were extended while crawling for each individual by treatment, total time (sec) each beetle was on its back by treatment, percent of non-overlapping area occupied by the detected beetle that changes between seconds, and beetle weight and sex. The most parsimonious models were compared and ranked by corrected Akaike’s Information Criterion (AICc) values, which limits overparameterization [46]. Likelihood ratio tests (LRT) were used to compare the top models with an AIC_*wt*_ ≥0.1 (ɑ  =  0.05). A final set of GLMMs determined if there was an interaction among treatment levels and the fixed effects identified in the top model (i.e., treatment * recorded behavior and movement parameters). A random effect of beetle identity was included in each GLMM.

## Results

### Measured exposure concentrations

Mean measured imidacloprid-spiked acetone solutions were 96.1% of the target nominal doses: 60, 120, and 240 ng/μL (nominal) and 52.8 ± 1.0, 113.3 ± 5.7, 254.1 ± 3.4 ng/μL (measured). Acetone used for the method blank and control groups had no detections of imidacloprid (LOD < 0.0025 ng/μL). Chemical analyses included metabolites (imidacloprid olefin, imidacloprid urea, imidacloprid 5-hydroxy), which were also below the LOD. All calculated endpoints were based on measured concentrations.

### *Nicrophorus* spp. dose-response contact toxicity

Calculated LD_X_ values are presented in Table 1. *Nicrophorus* spp. did not display 100% mortality within concentration-series, measuring 77 ± 9% mortality at the highest dose (nominal concentration 240 ng/beetle). One control beetle (*N*. *carolinus*) topically exposed to acetone-only died. Mean *N*. *marginatus* and *N*. *carolinus* weight was 0.44 ± 0.02 g (*n =* 73) and 0.84 ± 0.04 g (*n* = 47), respectively. The interaction between *Nicrophorus* spp. (i.e., *N*. *marginatus* and *N*. *carolinus*) and imidacloprid dose was not statistically significant (β ± SE  =  -2.7 ± 4.0, *p*  =  0.504), but the interaction between *Nicrophorus* spp. weight and imidacloprid dose was significant (β ± SE =  16.8 ± 0.002, *p* < 0.001), where heavier beetles (regardless of species) displayed greater survivorship. Weight-corrected LD_X_ values are presented in Table 1.

**Table 1:** *Nicrophorus* spp. toxicity values.

LD_X_^a^	ng/beetle^b^ (95% CI)	ng/g^c^ (95% CI)
10	58.9 (39.4–88.1)	98.2 (65.7–146.8)
20	83.0 (61.7–111.7)	138.3 (102.8–186.2)
50	149.3 (119.3–186.9)	248.8 (198.8–311.5)

Calculated *Nicrophorus* spp. contact toxicity values 24 hours after exposure to technical grade imidacloprid.

^a^Concentrations estimated to produce 10%, 20%, and 50% mortality ± 95% confidence intervals (CI).

^b^Average *Nicrophorus* spp. weight 0.6 ± 0.03 g (*n* = 120).

^c^Weight-corrected LD_X_ values.

### *Nicrophorus* americanus imidacloprid-induced behavior and mortality

The LD10 imidacloprid contact toxicity value (95% CI) for *Nicrophorus* spp. was 58.9 (39.4–88.1) ng/beetle. Experiments were conducted before chemical analysis, and *N*. *americanus* LD10 treatments were dosed with 1 μL of the 60 ng/μL nominal concentration. LC–MS/MS determined the measured mean (± SE) concentration was 52.8 ± 1 ng/μL (*n* = 3), approximately 88% of the nominal and 6.1 ng less than the target LD10 dose.

Mean (± SE) EthoVision-XT tracking efficiency was 98.5 ± 1.5% among all videos used. Two control *N*. *americanus* videos did not meet the duration criteria or were lost. Total distance (*t*(25) = -2.80, *p* = 0.03) and mean velocity (*t*(25) = -2.82, *p* = 0.02) were significantly different between the control and single LD10 dose. Between the control and repeated LD10 dose, total distance (*t*(25) = -1.81, *p* = 0.19) and mean velocity (*t*(25) = -1.93, *p* = 0.15) were not significantly different (Table 2).

**Table 2:** *N*. *americanus* movement and behavior parameters.

Treatment	Distance (mm)	Mobility (%)	Velocity (mm/sec)	High predation risk behavior (sec)	
				Flipped over	Wings extended
Control^a^	1977 ± 491	14.8 ± 2.9	3.3 ± 0.8	0 ± 0	9 ± 8
Single dose^b^	8593 ± 2108*	43.8 ± 5.8*	14.3 ± 3.5*	56 ± 24	146 ± 54
Repeated dose^b^	6241 ± 1514	37.9 ± 5.7*	10.9 ± 2.5	72 ± 38	114 ± 58

Mean (± SE) values of behavior and movement parameters estimated for each LD10 imidacloprid treatment group with *Nicrophorus americanus*. Asterisks denote significance between treatment groups and the control (*p* < 0.05, ANOVA followed by post-hoc Tukey pairwise comparison).

^a^Eight replicate beetles.

^b^Ten replicate beetles.

**Fig 1:**
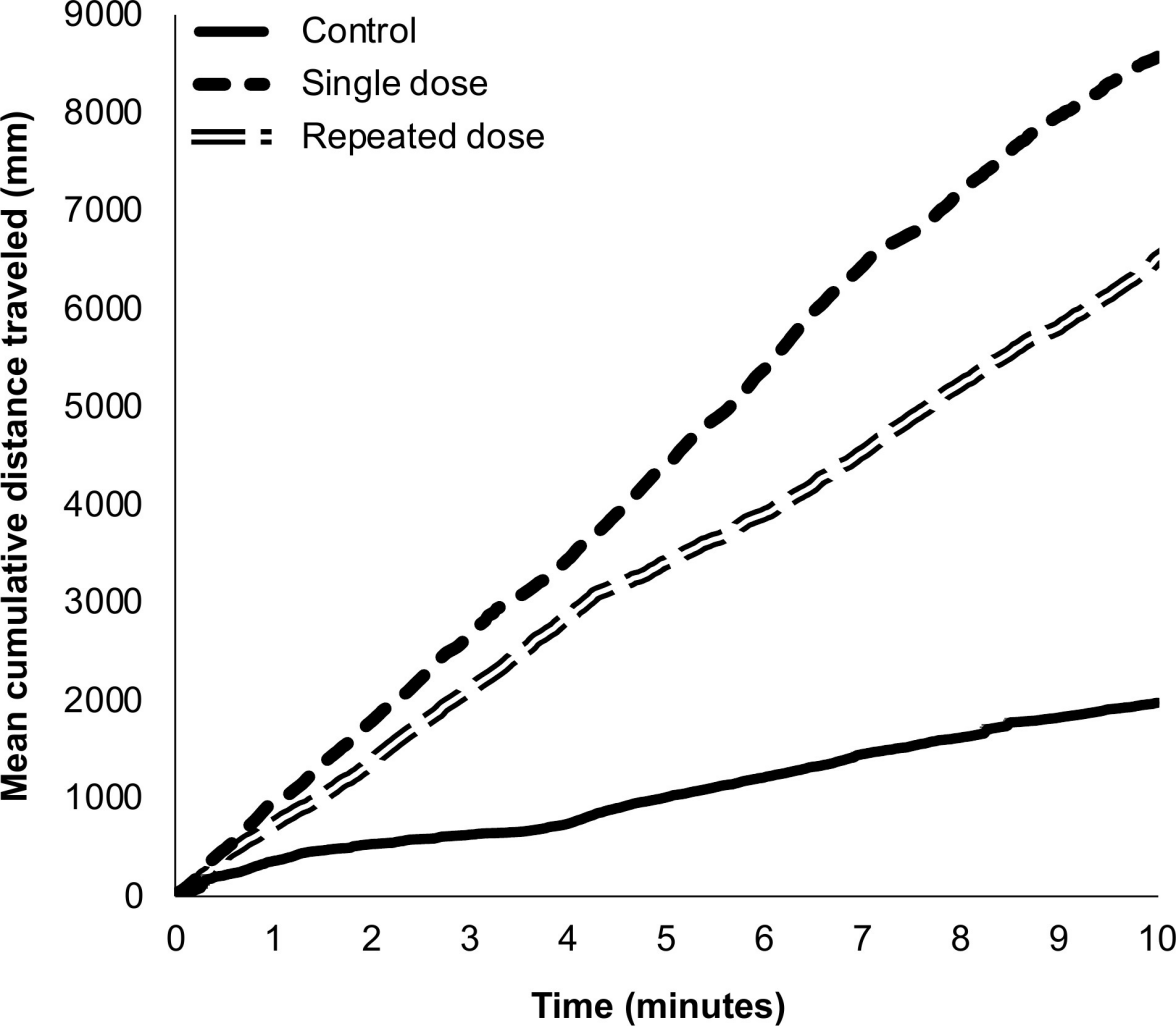
*americanus* distance traveled. ***N*.** Mean cumulative distance traveled (mm) by *N*. *americanus* over the 10-minutes post-dose observation period from each LD10 imidacloprid treatment group. Variation in distance per second among treatments was comparable (range in SE): Control (0.1–22.8 mm), single dose (0.8–14.6 mm), and repeated dose (0.9–15.2 mm).

**Fig 2:**
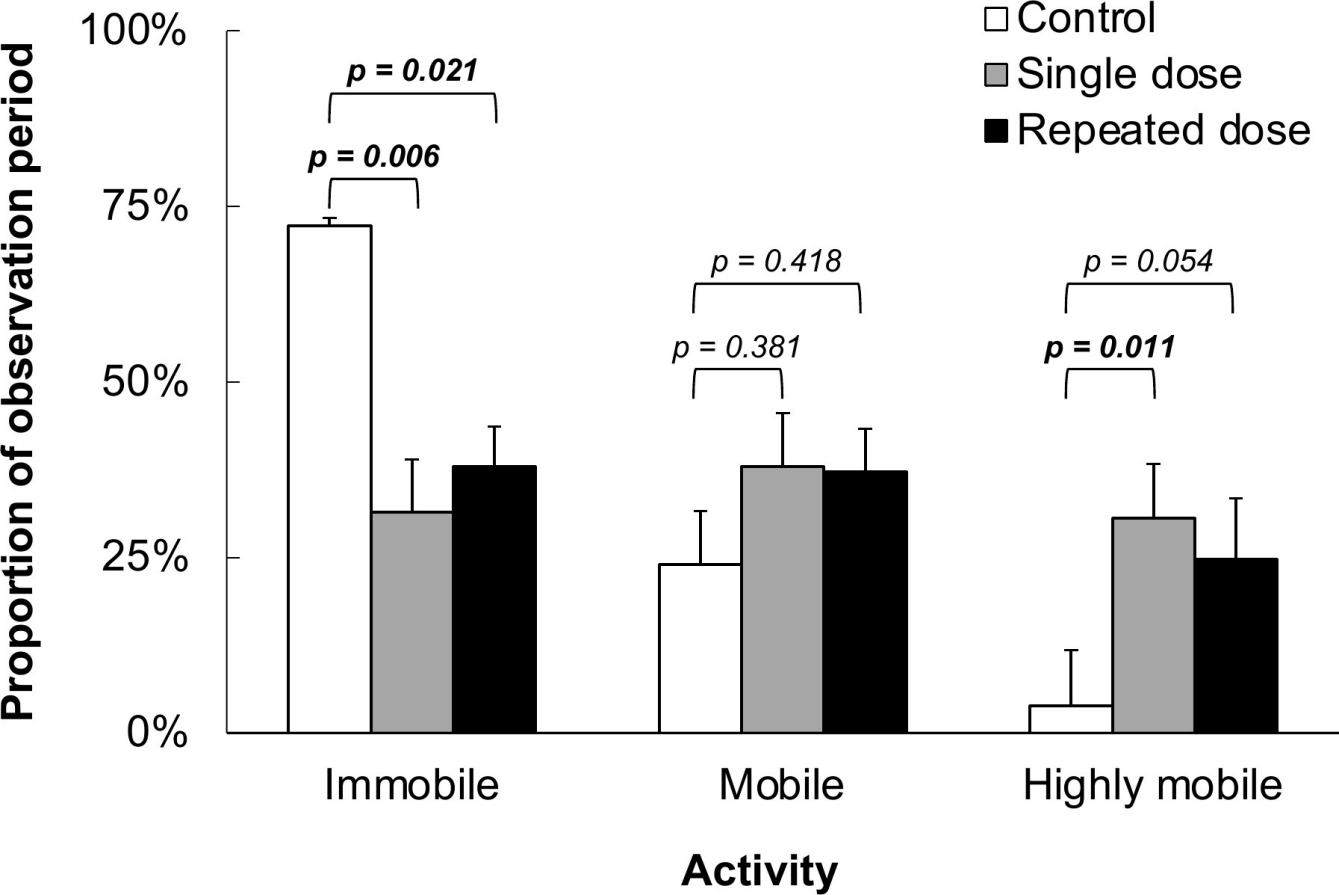
Mobility status among treatments. Comparison of mobility status graded by % activity (immobile: <20%, mobile: 20% - 60%, highly mobile: >60%) among three treatment levels, i.e., control, single LD10 dose, and repeated LD10 dose, evaluated by pairwise comparisons to the control using posthoc Tukey tests (ɑ  =  0.05).

On average (± SE), single LD10 dose *N*. *americanus* traveled 8593 ± 2108 mm, further than other treatment groups over time (Fig 1). Single LD10 dose (*t*(25) = -3.81, *p* = 0.002) and repeated LD10 dose (*t*(25) = -3.03, *p* = 0.02) *N*. *americanus* were more mobile (% mobility) than the control group (Table 2). In the LD10 dose treatment group, the time of beetle wing extension and the time each beetle was on its back were greater, but were not significantly different when compared to the control group (*p* > 0.05). However, time beetle wings were extended after a single LD10 exposure may have some utility as a behavioral indicator for exposure (*t*(25) = -2.45, *p* = 0.054). No significant differences were determined for all movement and behavioral parameters between single LD10 dose and repeated LD10 dose (*p* > 0.05). Compared to both LD10 dose treatments, the control *N*. *americanus* were significantly more immobile (Fig 2), where individuals exhibited death-feigning (tonic immobility), a common anti-predator behavior. Conversely, *N*. *americanus* exposed to LD10 dose treatments were highly mobile, with the single LD10 dose *N*. *americanus* exhibiting highly mobile activity 30.6 ± 7.7% of the 10-minute observation period.

GLMMs were used to assess the relative sources of variation in movement and behavioral parameters that resulted in 10-day *N*. *americanus* mortality (Table 3). The top model included the fixed effects: total distance + mean velocity + wings extended, which accounted for 55.8% (adj. *R*^*2*^) of the variation. Only wings extended (vulnerable behavior) was the second model and accounted for 30.7% (adj. *R*^*2*^) of the variation, but the addition of total distance + mean velocity explained significantly more variation (likelihood ratio test [LRT]; χ2(2) = 7.07, *p* = 0.03).

**Table 3:** GLMM results for movement and behavioral parameters.

Model structure	Model parameters ^a^				
	AIC_*c*_	ΔAIC_*c*_	AIC_*wt*_	-2LL	adj. *R*^*2*^
Distance + Velocity + Wings extended	32.4	0.00	0.18	-9.84	0.558
Wings extended	33.7	1.34	0.10	-13.38	0.307
Intercept-only (null)	38.0	5.57	0.01	-16.75	<0.001

Top GLMM selection results (i.e., ranked AIC_*c*_) assessing the relative sources of variation in movement and behavioral parameters that resulted in 10-day *Nicrophorus americanus* mortality. Models with an AIC_*wt*_ ≥0.1 and the null (intercept-only) is listed. Beetle identity was included as a random effect for each model.

^a^Model parameters are listed by column: Corrected Akaike’s Information Criterion (AIC_*c*_), change in AIC_*c*_ (ΔAIC_*c*_), model weights (AIC_*wt*_), -2LL (−2 × log likelihood), and adj. *R*^*2*^ (adjusted R-squared). Covariates listed in global model structure: Distance (total distance [mm] each beetle traveled by treatment), Velocity (Mean velocity [mm/s] calculated from each beetle for the 10-minute observation period by treatment), Wings extended (total time beetle wings were extended while crawling for each individual by treatment), Flipped over (total time each beetle was on its back by treatment), Mobility (Percentage of non-overlapping area occupied by the detected beetle that changes between samples), beetle weight and sex.

Mean male and female *N*. *americanus* weight was 1.09 ± 0.06 g (*n* = 17) and 1.27 ± 0.07 g (*n* = 13), respectively. Weight (χ2(2) = 2.72, *p* = 0.09) and sex (χ2(2) = 1.56, *p* = 0.2) did not significantly influence *N*. *americanus* 10-day mortality by treatment. Feeding was not impacted by single LD10 (*t*(27) = -1.48, *p* = 0.3) or repeated LD10 doses (*t*(27) = -0.71, *p* = 0.7) compared to the controls. On average (± SE), beetles consumed 3.0 ± 0.2, 2.8 ± 0.2, and 3.1 ± 0.2 larvae per feeding for control, single LD10, and repeated LD10 treatments, respectively. All control *N*. *americanus* survived 10 days post-dose, and single LD10 and repeated LD10 exhibited 30% and 50% mortality, respectively (Table 4).

**Table 4:** *N*. *americanus* mortality.

Treatment	Day										Cumulative mortality
	1	2	3	4	5	6	7	8	9	10	
Control	0	0	0	0	0	0	0	0	0	0	0%
Single dose	0	0	0	0	0	0	2	0	1	0	30%
Repeated dose	0	1	0	0	2	0	2	0	0	0	50%

Daily number of dead *N*. *americanus* over time from each LD10 imidacloprid treatment group.

## Discussion

Although the potential threat from nontarget exposure to neonicotinoids is widely acknowledged, no study has evaluated the toxicity of neonicotinoid active ingredients on burying beetles, specifically the federally threatened *N*. *americanus*. Here, the threshold concentrations for contact toxicity of imidacloprid to *Nicrophorus* spp. congeners were within the bounds of residual neonicotinoid concentrations detected in airborne and settled particulate matter from planter exhaust and cattle feedlots [4,47–50]. Over a ten-day period of monitoring, *N*. *americanus* experienced 30% mortality after a single exposure to the nominal LD10 and repeated exposure caused an increase to 50% mortality, supporting time-cumulative toxicity of this class of insecticides as reported by Sánchez-Bayo and Tennekes [8]. Beyond mortality, our data demonstrate significant behavioral alterations after a single and repeated LD10 application of imidacloprid, which may put *N*. *americanus* at greater risk of starvation, desiccation, or predation. We acknowledge the limitations of extrapolating laboratory-based bioassays to potential field exposure scenarios (i.e., direct dosing vs. active ingredient bound to dust or soil particles). However, similar standardized assessments are commonly used to characterize potential impacts of insecticides to other insect taxa, and this study provides context for future work with burying beetles. Behavioral responses linked to environmentally relevant exposure concentrations can provide further insight to environmental risk assessment, and our study highlights the potential threat of neonicotinoids to *Nicrophorus* spp., which exhibit unique behavioral characteristics and complex life history strategies.

Behavior is fundamentally linked to individual fitness, which may influence population-level effects, and is a sensitive indicator of pesticide exposure [51]. As cholinergic neuron agonists, neonicotinoids evoke repeated neuronal currents, prompting hyper-excitation of neurons for extended periods [52]. Electrophysiological activity of neonicotinoids and subsequent behavioral effects are well-studied in honeybees [11]. Previous research on neonicotinoid-induced behavioral impairment also includes ground beetles [53], bumblebees [54], and locusts [55]. In the present study, behavioral responses of imidacloprid-exposed beetles were significantly different from the controls, with a single LD10 dose prompting greater distance traveled and at greater velocity than repeated LD10 doses (Table 2). The carabid beetle, *Platynus assimilis* Paykull, had a similar hyperactive locomotor response post-ingestion of thiamethoxam at low doses, ranging from 0.04 to 4 ng/beetle [53]. Vincent et al. [56] documented an increase in angular speed and decrease in linear speed in the ladybird beetle larvae, *Harmonia axyridis* Pallas, post-contact to glass surface treated with 0.3 μg imidacloprid/μL, suggesting greater erratic movement. After exposure to imidacloprid, the ground beetle, *Harpalus pensylvanicus* Degeer, exhibited excessive grooming, impaired walking, and intoxicated behaviors that could lead to increased vulnerability to predation by ants [57]. For burying beetles, increased activity may expend limited nutrients acquired from feeding on patchy and ephemeral carrion resources [21]. Moreover, hyperexcitation will weaken the insect from exhaustion [52,53], leading to increased starvation, vulnerability to predation, or deficits in competition for breeding resources.

Recurrent neonicotinoid exposure results in the depolarization and inactivation of affected Na+ voltage channels, where action potential firing ceases and functionally desensitizes nAChRs [58]. Thus, repeated neonicotinoid exposure in succession can cause time-dependent effects [59], with some symptoms occurring in stages [53]. Chronic accumulation can reinforce toxic effects, including paralysis, shaking, exhaustion of cellular energy, and reduced mobility [9]. Here, we observed a decrease in distance traveled and velocity in repeated LD10 dosed *N*. *americanus* when compared to the single LD10 treatment (Table 2). Congruent between both the single and repeated LD10 treatments, distance traveled and velocity explained a significant amount of variation in mortality assessed by GLMMs (Table 3), confirming their sensitivity as behavioral indicators of toxicity. Transitioning from locomotor hyperactivity to locomotor hypoactivity is documented among neurotoxic compounds and can sometimes equally impact individual condition [60]. Regardless of dose, Tooming et al. [53] observed sluggish behavior following exposure of *P*. *assimilis* to thiamethoxam, which may impede predation success and rate. Timing and circumstances of exhibiting hyperactivity, hypoactivity, or atypical behaviors is important to put into a species-specific context.

Prior to carcass burial, adult burying beetles are particularly vulnerable to predation with documented consumption by vertebrate scavengers attracted to carrion [17,21]. Immediately after exposure to a single LD10 or repeated LD10 dose, *N*. *americanus* displayed behaviors that may put them at higher risk to predation. During the 10-minute observation period (expressed here as % of time), imidacloprid-exposed beetles struggled to maintain an upright posture (9–12% flipped over) and extended their wings (19–24%) without taking flight. Extended wings explained 30.7% of the variation and was identified in both top model sets as a potential predictor of mortality (Table 3). Conversely, control *N*. *americanus* were mostly immobile after being dosed with acetone (control treatment), barely crossing the diameter of the entire arena (Fig 1). Death-feigning, or tonic immobility, is used by burying beetles to avoid predation [61]. All individuals were handled just before video recording, which likely simulated a predation event, and should have uniformly prompted death-feigning behavior. However, the inability to maintain controlled, coordinate movements or remain immobile under natural conditions may influence predation risk. Vulnerability of ground beetles after sublethal neonicotinoid exposure has been reported under laboratory and field conditions [53,57,62]. With demonstrated population-level effects at sublethal concentrations, behavioral ecotoxicology is increasingly recognized as an underdeveloped regulatory step towards more comprehensive pesticide risk assessment [51].

Potential neonicotinoid use in the existing range of *N*. *americanus* is categorized as “high” and mostly restricted to field crops [34], but considerable segments of its range also overlap with livestock operations [29]. Both types of agricultural operations emit comparable amounts of neonicotinoid-bound fugitive particulate matter [4,47,50]. Measurable levels of neonicotinoids have been detected near conventional farms on the soil surface, in vegetation, and in insects [4,5,7,49]. Wind conditions play a significant role in off-site mobility of particulate matter, transporting active ingredients to nontarget habitats [63], traveling approximately 12 km downwind in some instances [64]. Neonicotinoid-bound particulate matter in air 1 to 9 meters into vegetative field buffers near maize planting ranged between 200 to 1600 ng/m^3^, which yielded concentrations of 71 to 434 ng/caged bee [4]. In comparison, Peterson et al. [50] measured up to 1125 ng/m^3^ from beef feedlots, translating to approximately 13.3 × 10^9^ ng/day.

For standard insects used in pesticide risk assessment, the contact LD50 values are 60 ng/bee for honey bees (*Apis mellifera* L.) and 20 ng/bee for bumble bees (*Bombus* spp.) [65]. Forero et al. [66] measured neonicotinoid-bound particulate matter from field edges, which amounted to 1.1 to 36.4% of the reference honeybee LD50; these values track with similar studies in North America, estimating honeybees encounter 2.7 to 28 ng/bee of neonicotinoid residues in seed dust drift [67]. Collectively, previous studies have demonstrated the potential for frequent interaction with sublethal neonicotinoid concentrations. There is limited baseline pesticide toxicity data for beetles in general and for beetles of conservation concern.

Here, imidacloprid demonstrated measurable acute (24-hour) toxicity to adult *Nicrophorus* spp. (Table 1), with an LD50 of 149.3 ng/beetle. We did not observe a similar response in 24-hour toxicity between *N*. *americanus* and *Nicrophorus* spp. congeners. We observed no *N*. *americanus* mortality until Day 7 in the single LD10 dosed group. However, with a limited sample size of *N*. *americanus*, we observed 30% mortality by Day 10 in individuals exposed to a single LD10 dose of 52.8 ng/beetle (Table 4). Importantly, the average weight of the *Nicrophorus* spp. use to generate the acute contact toxicity values was 0.6 ± 0.03 g (*n* = 120), whereas *N*. *americanus* weighed nearly double the mean *Nicrophorus* spp. weight at 1.18 ± 0.05 g (*n* = 28). Importantly, the interaction between *Nicrophorus* spp. weight and imidacloprid dose was significant, where heavier beetles (regardless of species) displayed greater survivorship. Normalized for weight, the ng/beetle LD50, LD20, and LD10 values listed in Table 1 are 248.8, 138.3, and 98.2 ng/g, respectively. Accordingly, the weight-corrected LD10 dose for *N*. *americanus* should have been 115.8 ng/beetle; however, we did not determine this until after conducting the behavioral assays. Here, the weight-corrected dose applied to *N*. *americanus* was approximately equivalent to an LD5.

Using similar acute (24-hour) contact application methods, Cavallaro et al. [16] determined telecoprid dung beetles to be extremely sensitive to imidacloprid with an LD50 of 19.1 ng/beetle, demonstrating that dung beetles may be more sensitive to imidacloprid than burying beetles. Taxa-specific sensitivity to imidacloprid has been previously documented between two predatory beetle species, where a soldier beetle, *Chauliognathus flavipes* Hentz, was ten times more sensitive than the spotless ladybird beetle, *Cycloneda sanguinea* L., exposed to insecticide-treated filter paper disks [68]. Using neonicotinoid-coated vials, Svehla et al. [36] reported elevated toxicity of imidacloprid to *Cicindela circumpicta* larvae (first instar) with an LD50 of 0.23 ng, which was used as a surrogate species for the endangered Salt Creek tiger beetle, *Cicindela nevadica lincolniana* Casey. The comparative toxicity and applicability of congeners, surrogate species, or model beetle taxon needs to be further explored to characterize risks of nontarget pesticide exposure.

Recent spatial analyses that used acute honeybee toxicity values (i.e., contact and oral LD50) found a multi-fold increase in the toxic loading of insecticides in the United States driven by neonicotinoid application [69,70]. Regionally critical areas for *N*. *americanus*, the Northern Great Plains and Prairie Gateway, showed more than a 30-fold and 10-fold increase in toxic load from 1997 to 2012, respectively [70]. In the Midwest, habitat continuity among areas not affected by neonicotinoid exposure is limited, and the fragmentation of *N*. *americanus* range may be further compounded by barriers of pesticide exposure during planting. For example, a state-wide assessment of seed dust drift estimated that over 94% of foraging honey bees are at risk of neonicotinoid exposure at varying concentrations during sowing of corn in Indiana [67]. As a habitat generalist, *N*. *americanus* can be found in grasslands, wet meadows, and partially or completely forested areas [71], and much of their extant range is peripheral or embedded in agricultural matrices [34]. Leasure and Hoback [29] described a negative spatial relationship between extant *N*. *americanus* distribution and pesticide application rates associated with cropland. However, conventional agricultural operations prompt multiple physical and chemical stressors (i.e., planting and harvest, fertilizer and pesticide applications) on nontarget habitats that can be spatially and temporally correlated, challenging interpretation.

Regionally-specific modeling approaches that account for total mass of applied insecticides and *N*. *americanus* range paired with baseline toxicity data can better inform species-specific risk assessment. Converging previous habitat suitability modeling efforts [71] and vastly improving existing efforts such as the US EPA Magnitude of effect tool (MAGtool v2.4; [72]) are among the future directions that could be explored. Important to note, the MAGtool was designed to help determine the magnitude of potential pesticide use on threatened or endangered species, and currently, it does not include potential exposure of neonicotinoid-treated seeds to terrestrial invertebrates. To adequately protect listed insect species, like *N*. *americanus*, future efforts must incorporate seed treatment use to scale the potential threat it poses.

Considering the likelihood of nontarget pesticide exposure under field conditions, ecological selectivity may result from differences in life history strategies, pesticide fate and distribution, or other environmental factors. With the exception of diurnally active *Nicrophorus* spp. (including *N*. *carolinus* and *N*. *marginatus*), aerial dusting directly on burying beetles is unlikely, especially for nocturnal species, including *N*. *americanus*. USFWS [32] correctly asserts that *N*. *americanus* spends the majority of its life cycle underground, limiting exposure to direct contact with aerial pesticide applications. Within its range, *N*. *americanus* is associated with sandy loam soils and higher moisture, i.e., wetlands, topographical wetness, greater mean annual precipitation [29,71]. In agricultural watersheds, neonicotinoids, which are highly water soluble, are used as systemic seed treatments where active ingredients are readily dissolved and mobile in the environment via soil pore water and groundwater [73–75]. Soil may temporally act as a sink for neonicotinoids before potential runoff events transport active ingredients to nearby surface water [74]. In the United States, residual neonicotinoid concentrations have been measured in soils sampled in the Midwest and Southern states [7,49,76,77].

Dispersal of seed dust on surface soils in field margins near corn and soybean fields in Indiana measured 2.9–7.3 ng of imidacloprid/g of soil (dry weight) [49]. Main et al. [7] report similar values of clothianidin in nontarget field margins at <3 ng/g in Missouri. More recent surveys found total neonicotinoid concentrations in surface soils from 2.1 to 4.9 ng/g in Illinois conservation areas near planting and can measure over 3 ng/g in soils 10–20 cm deep [78]. Soil characteristics and moisture are critical factors that influence *N*. *americanus* occurrence [21]. Depending on the soil characteristics and light exposure, neonicotinoid active ingredients can bind and persist in soil for months or years [73,79], potentially leading to incidental soil contact and soil-water ingestion by burying beetles. Given the sensitivity of *N*. *americanus* to topical neonicotinoid application, future studies should examine the chronic toxicity of neonicotinoids to burying beetles under field-realistic concentrations, focusing on daily burial, reproductive success, and mortality of larvae and pupae after chronic neonicotinoid exposure in soil.

## Conclusions

Drivers of *N*. *americanus* declines include landscape-level habitat loss and fragmentation, competition and carrion resource dynamics, and potential nontarget exposure to pesticides [28]: a cumulative product of agricultural intensification. Prior to *N*. *americanus* being listed as an endangered species, researchers suggested that the widespread use of dichlorodiphenyltrichloroethane (DDT) may have initially contributed to steep population declines [35]. Since then, few studies have discussed or even considered the toxicity of specific insecticide active ingredients to *N*. *americanus* [80]. Our data provide a clear precedent for further hypothesis testing of different neonicotinoid exposure profiles to all life stages of *N*. *americanus*, specifically chronic soil exposure and contaminated carrion, i.e., seed treatment poisoning of small mammals and birds [81]. The exploration of more complex behavior endpoints, i.e., movement, anti-predator behavior, carcass seeking, overwintering, interspecific competition, and parental care, should also be considered. Using congener species or model beetle taxon (e.g., Carabidae, Staphylinidae, Tenebrionidae), screening the relative toxicity of other pesticide classes must be addressed to better protect *N*. *americanus* from nontarget exposure. Under the Federal Insecticide, Fungicide, and Rodenticide Act, the US EPA is responsible for ensuring that registered pesticide use does not cause unreasonable adverse effects on the environment, including listed species and their critical habitats; however, the US EPA has yet to meet the Endangered Species Act criteria for over 95% of all registered pesticides [82].

## Supporting information

S1 Table *Nicrophorus* spp. LD data. (XLSX)

S2 Table *Nicrophorus americanus* mortality and behavior data. (XLSX)

S3 Table *Nicrophorus americanus* food consumption data. (XLSX)

S4 Table Extracted *Nicrophorus americanus* distance (mm) data from EthoVision-XT. (XLSX)
